# Flavonoids as Antiviral Agents for *Enterovirus A71* (*EV*-*A71*)

**DOI:** 10.3390/v12020184

**Published:** 2020-02-06

**Authors:** Salima Lalani, Chit Laa Poh

**Affiliations:** Centre for Virus and Vaccine Research, Sunway University, Bandar Sunway, Subang Jaya, Selangor 47500, Malaysia

**Keywords:** flavonoids, antivirals, *Enterovirus 71 (EV*-*A71)*, infectious childhood disease, hand, foot and mouth disease (HFMD), viruses

## Abstract

Flavonoids are natural biomolecules that are known to be effective antivirals. These biomolecules can act at different stages of viral infection, particularly at the molecular level to inhibit viral growth. *Enterovirus A71 (EV*-*A71)*, a non-enveloped RNA virus, is one of the causative agents of hand, foot and mouth disease (HFMD), which is prevalent in Asia. Despite much effort, no clinically approved antiviral treatment is available for children suffering from HFMD. Flavonoids from plants serve as a vast reservoir of therapeutically active constituents that have been explored as potential antiviral candidates against RNA and DNA viruses. Here, we reviewed flavonoids as evidence-based natural sources of antivirals against non-picornaviruses and picornaviruses. The detailed molecular mechanisms involved in the inhibition of *EV*-*A71* infections are discussed.

## 1. Introduction

Flavonoids are naturally occurring polyphenolic biomolecules widely found in plants and are responsible for a wide variety of biological functions [1]. Pharmacological properties of flavonoids include antioxidant, anti-inflammatory, anticancer, antimicrobial and immunomodulatory functions [2]. More than 6000 compounds have been identified as flavonoids based on their basic structures consisting of C6-C3-C6 and they are divided into several classes such as flavonols, flavanones, isoflavones, flavones, and anthocyanidins. Due to a wide range of biological activities being exhibited by flavonoids, they have become molecules of interest for natural drug discovery research. In this review, antiviral mechanisms of flavonoids, mainly against *Enterovirus A*-*71 (EV*-*A71)*, will be discussed to provide evidence-based efficacy of antiviral properties.

## 2. Flavonoids against Viruses

Flavonoids have been studied against a wide range of DNA and RNA viruses. In general, flavonoids work by several mechanisms. They can block attachment and entry of viruses into cells, interfere with various stages of viral replication processes or translation and polyprotein processing to prevent the release of the viruses to infect other cells. Different flavonoids have been found to inhibit the virus through various mechanisms. Based on antiviral mechanisms of action, flavonoids can be prophylactic inhibitors, therapeutic inhibitors or indirect inhibitors by interaction with the immune system. Flavonoids that inhibit viral activity can be further divided into the following sub-categories.

- Flavonoids that bind to specific extracellular regions of the virus such as viral proteins present on the capsids.
- Flavonoids that prevent attachment or entry of the virus into host cells. In some cases, flavonoids can bind to virions and modify the virus structure. Though the virus can still internalize, the process of viral uncoating is stalled.
- Early-stage replication inhibitors.
- Transcription and translation blockers.
- Inhibition of late stages of maturation such as inhibition of assembly/packaging and release.
- Flavonoids that can inhibit viral infections by interfering with host factors that are required for successful infection or modulating the immune system to reduce the viral load.

### 2.1. Flavonoids against Non-Picornaviruses

Research has been carried out on plants that are being used in traditional medicines in different parts of the world. Many natural compounds of various chemical classes have been identified as potential therapeutic agents. Several flavonoids have been studied to evaluate their antiviral potential. For example, quercetin and quercitrin were first demonstrated by Cutting et al. (1949) to prophylactically inhibit rabies virus in mice [3]. Moreover, quercetin was found to exhibit its antiviral activity against *vesicular stomatitis virus (VSV)* by activation of macrophages [4]. Biologically active components of Elderberry extract dihydromyricetin and tetra-*O*-methyl quercetin were found to inhibit *influenza A virus* in vitro, most likely by binding to viral mannose-rich hemagglutinin domains which are important for the virus to enter the host cells [5]. Mechanistic studies later showed that it was able to inhibit neuraminidase activity of *influenza A virus* by interaction with the subunit 2 of the hemagglutinin that resulted in virus entry inhibition [6]. Quercitrin, a rhamnoside derivative of quercetin, was found to inhibit the initial stage of viral replication of the *influenza A virus*, evident by a decrease in mRNA synthesis of the virus. The study also concluded that quercitrin was unable to interact directly with virus particles [7]. Quercetin was shown to inactivate the NS3 helicase and NS5 protease of *hepatitis C virus (HCV)* [8,9]. Quercetin also blocked viral binding and entry of *herpes simplex virus (HSV)*-*1, HSV*-*2,* and drug-resistant *HSV*-*1* to Madin–Darby canine kidney NBL-2 (MDCK) cells [6]. Another flavonoid derivative, 3-β-*O*-d-glucoside, was able to protect mice from *Ebola virus* infection when given prior to virus challenge. However, the mechanism by which this flavonoid inhibited viral entry is unclear [10].

In a similar manner, sulfated rutin was shown to block the entry of *human immunodeficiency viruse (HIV)*-*1* without interactions with the host cell membrane. Cell fusion and entry assays revealed that sulfated rutin could drastically inhibit *HIV*-*1* infection when cells were treated during the early adsorption phase. The probable mechanism proposed by investigators was the inhibition of *HIV* glycoprotein-mediated cell-cell fusion step [11]. In the same study, it was identified that sulfated rutin could also inhibit *HSV*, the mechanism of inhibition is still unknown.

Apigenin was reported as an antiviral against *HCV* by host factor modulation. It caused a reduction in mature miRNA122 production that regulated *HCV* infection in vitro [12]. Baicalin was found to interfere with the interaction between the *HIV*-*1* envelope protein and host immune cells. This anti-*HIV* activity was achieved by inhibition of the *HIV*-*1* envelope glycoprotein (gp120)-mediated fusion with T cells and monocytes expressing CD4/CXCR4 or CD4/CCR5 [13]. In addition, baicalin was also reported to inhibit *dengue virus (DENV*-*2)* by the virucidal mechanism. It blocked the attachment of *DENV*-*2* to the Vero cells but did not show any activity during the virus entry stage [14]. Baicalin was able to induce the production of interferon-gamma (IFN-ɣ) in natural killer (NK), CD4+ and CD8+ T cells during in vitro *influenza A virus* infection. It was suggested that baicalin was able to directly bind NS1–p85β (RNA binding domain), which was the main mediator to downregulate IFN-ɣ [15]. The induction of IFN-ɣ triggered the immune system to activate the Janus kinase/signal transducer and activator of transcription protein 1 (JAK/STAT1) pathway, which led to the expression of *IFN*-*ɣ*-inducible genes. This expression of *IFN*-*ɣ* genes and signaling pathway facilitated the reduction of viral loads by immune modulation [16]. Baicalein, the parent compound of baicalin, has also been reported as antiviral against *Japanese encephalitis virus (JEV)* and *DENV*-*2*. It is postulated that baicalein inhibited *JEV* by probable interaction with structural proteins of virus and this prevented entry of the virus into the cells [17]. However, the antiviral mechanism against *DENV*-*2* was identified as replication inhibition in silico by interaction with viral NS3/NS2B and NS5 proteins [18].

Flavonoids, genistein and ginkgetin, were reported to inhibit assembly and release of *HIV* and *influenza A virus*, respectively. Genistein inhibited Vpu protein that is involved in the formation of ion channels in infected cells and thus controlled the release of *HIV* [19]. Ginkgetin inhibited sialidase activity of *influenza A virus* and thus inhibited virus assembly and release [20]. Fisetin possessed post-infection anti-*CHIKV* activity by the inhibition of NS proteins 1 and 3 in a dose-dependent manner. Moreover, it was observed that *CHIKV* E2 protein and its precursor pE2 were also downregulated during treatment with fisetin [21]. Similarly, a decreased replication process of *DENV*-*2* was observed when Vero cells were treated with fisetin after *DENV*-*2* infection. It was postulated that this antiviral activity could be due to the direct binding of fisetin to the viral RNA, thus impeding polymerase activity [22]. Kaempferol and its structural flavonol derivatives were studied to identify the suitable pharmacophore to design antiviral against the *influenza A virus*. This study revealed that most of the flavonols with structural similarity with kaempferol were able to noncompetitively inhibit neuraminidase enzyme. However, the exact interacting partner is still unknown [23]. Moreover, kaempferol was also reported to inhibit 3a channels of coronavirus and blocked the release of virus progeny [24]. Luteolin was found to interfere with the entry of *influenza A virus* and *severe acute respiratory syndrome coronavirus (SARS*-*CoV)* by interacting with hemagglutinins of *influenza A virus* and S2 protein of *SARS CoV* viruses, respectively [25,26].

Another group of flavonoids, viz. methoxyflavone, isoscutellarein, and 8-methoxy-isoscutellarein, were reported to inhibit early replication of *influenza A virus* by reduction of sialidase activity, inhibition of lysosomal fusion and RNA polymerase activity [27]. Naringenin, a flavanone, also possessed antiviral activities against *HCV* [28], *DENV*-*2*, *DENV*-*4* [29] and *CHIKV* [30] by replication inhibition at RNA and protein levels. Other flavonoid such as silymarin which has a broad-spectrum antiviral activity [31,32,33] was able to block entry and fusion of *HCV* viral pseudo-particles (pp) when Huh-7.5 cells were pre-treated with silymarin [34]. Moreover, it was shown to interfere with the replication process of *DEN*-*V*, *CHIKV* and *influenza A virus* [35,36,37].

Tea catechins were also reported to inhibit the *influenza A virus* by binding to the hemagglutinins and restricted virus adsorption, preventing the penetration of the virus into the cells [38]. Tea catechins were studied in clinical trials by Matsumoto and co-workers (2011). They conducted a randomized controlled trial to study the effects of tea catechins in the prevention of influenza virus infections in healthcare workers in elderly homes. Tea catechins were effective in preventing the clinical incidence of influenza infections in the treatment group as compared to the placebo group [39]. Epigallocatechin (EGC), a tea catechin flavonoid, was reported to inhibit the replication of *influenza A* and *B viruses* by in vitro acidification of the lysosomal and endosomal environment through clathrin-mediated endocytosis [40]. Apart from having antiviral activity against the influenza virus, another flavonoid from tea catechins, epigallocatechin gallate (EGCG), was shown to directly bind to CD4+ T-cells. This binding blocked the binding of the *HIV*-*1* envelope protein (gp120) and caused viral entry inhibition [41]. EGCG was also demonstrated to bind to the glycoprotein B and glycoprotein D of *HSV* to render it non-infective [42]. Moreover, EGCG was shown to have potent antiviral activity against CHIKV by blocking its entry, probably by competing for cellular co-receptors of target cells such as heparan sulfate or sialic acid [43]. EGCG was also found to inhibit the replication of *Epstein–Barr virus (EBV)* by inhibition of *Zta*, *Rta* and *EA*-*D* genes. This inhibition interrupted the MEK/ERK1/2 and PI3-K/Akt signaling pathway of the lytic cycle EBV [44]. EGCG also inhibited *hepatitis B virus (HBV)* replication by impairment of the production of pre-core mRNA and replicative intermediates of DNA [45]. *Zika virus* was also inhibited by EGCG, as it was able to interact with the lipid envelope and blocked the entry of virus into cells [46]. It also caused acidification in lysosomes to make an unfavorable environment for virus replication [47]. The antiviral activities of several other flavonoids according to the mechanisms of inhibition against various viruses are summarized in Table 1. Structures of these flavonoids can be found in Supplementary material Table S1: Molecular structures of antiviral flavonoids.

### 2.2. Flavonoids against Non-EV-A71 Picornaviruses

Picornaviruses are non-enveloped RNA viruses belonging to the picornaviridae family. Their natural hosts are vertebrates such as reptiles, fish, amphibians, birds and mammals. Viral infections such as poliomyelitis, hepatitis A, common cold and hand, foot and mouth disease are caused by picornaviruses. Flavonoids, in particular, have shown promising results in the laboratory against picornaviruses. Some of these flavonoids are discussed.

Quercetin, a well-studied flavonoid, exhibited anti-rhinoviral (RV) activity by inhibition of the viral transcription and translation processes. Quercetin decreased endocytosis of *RV* and reduced phosphorylation of Akt (effector of phosphoinositol 3-kinase) when epithelial cells were pretreated with quercetin. In addition, it also reduced viral infection when added 6 h post-infection in vitro. Moreover, it also repressed interferon and interleukin-8 response, resulting in lower viral RNA and capsid protein production. Quercetin demonstrated similar effects in vivo with decreased viral loads and suppression of viral immune mediators [50]. Quercetin was shown to protect mice when challenged with the quercetin-treated *Mengo virus*. It was interesting to note that there was no antiviral activity detected when quercetin was tested in L-929 cells [3]. This observation led to the study of the role of interferon induction in antiviral activity. However, there was no significant detection of interferons in drug-treated mice. This resulted in speculating that the antiviral activity was achieved by macrophage activation instead of interferon induction. In the same study, quercetin was also found to inhibit *encephalomyocarditis virus (EMCV)* by macrophage activation [4]. A methyl derivative of quercetin was shown to inhibit *poliovirus* by blocking the synthesis of viral genomic RNA and proteins [51,52]. Similarly, trimethylquercetin was shown to inhibit the production of progeny *coxsackievirus B4 (CV*-*B4)* and protected the mice from death upon lethal challenge [53]. In another independent study, dihydroquercetin isolated from *Larix sibirica* was found to mediate the in vivo *CV*-*B4* infection by inhibition of ROS induced oxidative stress [54].

Similarly, luteolin and its derivatives have been studied against picornaviruses. For example, luteolin was found to inhibit the RNA synthesis of *Coxsackievirus A16 (CV*-*A16)* [55]. Moreover, it was also reported to inhibit *poliovirus* at replication stage. However, the mechanism of inhibition of *poliovirus* by luteolin is still unknown [56]. In contrast to luteolin, its derivative eupafolin was shown to inhibit *CV*-*A16* by inhibition of viral attachment. It was shown to reduce the IL-6 and RANTES which led to the inactivation of virus-induced upregulated the ERK1/2, c-Jun, and STAT3 signaling pathways [57].

In a similar manner, kaemferol and its derivatives were also identified to be potent inhibitors of picornaviruses. Methylkaempferol was reported to inhibit the late replication step of the *poliovirus*. The inhibition mechanism is attributed to the potential of this flavonoid to inhibit the positive-strand synthesis of viral RNA [58]. Furthermore, Kaempferol-3-*O*-[2″,6″-di-*O*-Z-p-coumaroyl]-β-d-glucopyranoside and its derivatives were identified as replication inhibitors of *Rhinovirus 1B (HRV*-*1B)* and *Coxsackievirus B3 (CV*-*B3)* in vitro [59]. The group of methoxy flavones were reported to inhibit *poliovirus*-*1* by downregulation of signaling pathway and apoptosis [60]. Furthermore, entry of rhinovirus was shown to be inhibited in silico by inhibition of the protein grid of virus [61]. Another flavonoid sakuranetin was also identified as a potent inhibitor of *rhinovirus*-*3* by its antioxidant properties [62]. Several other flavonoids were reported to inhibit the activity of various picornaviruses [60,63,64,65,66]. However, their exact molecular mechanisms of actions are still unknown. Examples of bioflavonoids from various chemical classes exhibiting antiviral activities against picornaviruses are summarized in Table 2. Structures of these flavonoids can be found in Supplementary material Table S1: Molecular structures of antiviral flavonoids.

## 3. Flavonoids that Target *EV*-*A71*

Hand, foot and mouth disease (HFMD) is a global childhood infectious disease. It is predominant in Asia, with annual outbreaks ranging from two to three million cases. *EV*-*A71* is one of the main etiological agents of HFMD, prevalent in children under the age of 6 years. *EV*-*A71* belongs to the Picornaviridae family and is classified as a non-enveloped RNA virus. The 7.4 kb genome of *EV*-*A71* encodes four structural viral proteins (VP1 to VP4) and seven non-structural proteins (2A to 2C and 3A to 3D). These structural and non-structural proteins are generally being considered for designing effective antiviral agents against *EV*-*A71*.

The life cycle of *EV*-*A71* initiates with binding to host receptors such as SCARB2, vimentin and annexin 2. Once attached, the virus enters into the cell through endocytosis and uncoating starts in an endosome. Thereafter, the translation of mRNA and polyprotein processing occur. Human hnRNP A1 is an interaction partner of viral internal ribosome entry site (IRES) whereas hnRNP A2 is highly similar to hnRNP A1. Upon interaction of IRES with hnRNP A1 and hnRNP A2, the viral translation process is activated. Structural and non-structural viral proteins are formed via cleavage by 2A and 3C proteases. This is followed by the replication and assembly of viral RNA into the capsid, maturation of viral particles and release of virus from the cells [69].

**Figure 1:**
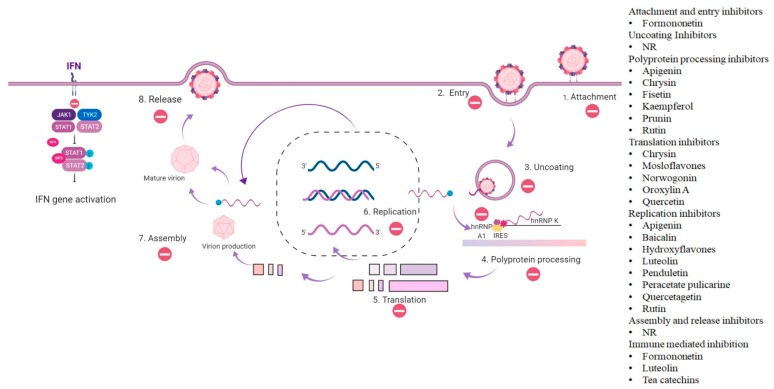
The antiviral activity of flavonoids in the life cycle of *EV*-*A71*. Flavonoids are reported to exhibit antiviral activity against *EV*-*A71* and they are categorized according to the mechanism of inhibition at different stages of the virus life cycle. NR: not reported (Flavonoids that affect uncoating, assembly and release of *EV*-*A71* are not reported). HnRNP = heterogeneous nuclear ribonucleoproteins, IRES = internal ribosome entry site, IFN = interferon, JAK = janus kinase, TYK = tyrosine kinase, and STAT = signal transducer and activator of transcription proteins.

**Figure 2:**
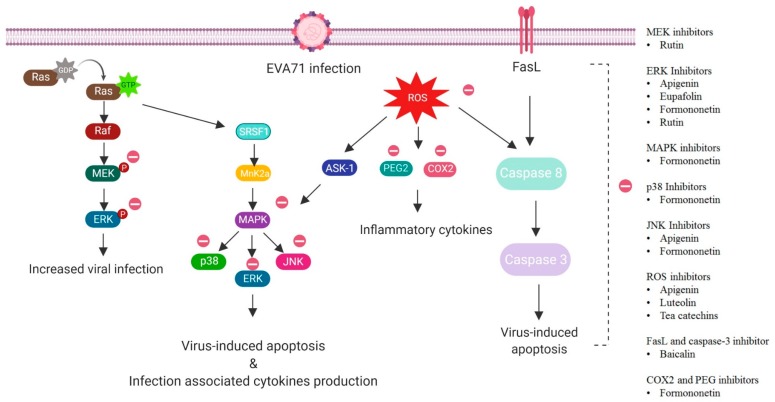
Inhibition of molecular signaling pathways involved in *EV*-*A71* infection by flavonoids. Flavonoids are classified according to their ability at the molecular level to inhibit signaling pathways involved in *EV*-*A71*-induced apoptosis, inflammation and infection associated cytokine production. GDP = guanosine diphosphate, GTP = guanosine triphosphate, RAF = rapidly accelerated fibrosarcoma, MEK/MAPK = mitogen-activated protein kinase, ERK = extracellular signal-regulated kinase, SRSF/MnK2a = serine/threonine-protein kinase. JNK = c-Jun N-terminal kinase, ASK = apoptosis signal-regulating kinase, PEG = prostaglandins, COX = cyclooxygenase, and FasL = Fas ligand.

Many flavonoids have been studied to identify their potential as antiviral agents against *EV*-*A71*. The majority of the flavonoids studied demonstrated the antiviral activity by interference with the *EV*-*A71* replication cycle (Figure 1). However, a few flavonoids have been reported to exert their antiviral activity by immune mediation (Figure 2). Examples of flavonoids with antiviral activities against *EV*-*A71* are discussed.

### 3.1. Apigenin

Apigenin is a flavone that has been tested for its antiviral potential against Fuyang and BrCr strains of *EV*-*A71*. It was able to reduce infection at the inhibitory concentration (IC_50_) of 10.3 μM. Apigenin inhibited *EV*-*A71* infection by suppressing viral internal ribosome entry site (IRES) activity. Apigenin was found to disrupt the viral RNA association with hnRNP A1 and A2 proteins [70].

Moreover, apigenin was found to inhibit cellular apoptosis by caspase-3 cleavage, which is considered as a prime process to release viral progeny. It is also a known scavenger of reactive oxygen species (ROS) and was found to reduce infection-induced ROS generation in *EV*-*A71* damaged cells. Furthermore, it decreased the cytokine levels involved in infection. Apigenin was confirmed to inhibit the activation of the c-Jun N-terminal kinase (JNK) pathway required for the replication process and release of progeny *EV*-*A71*. Another downstream p38 MAP kinase signaling pathway was found to be partially inhibited by apigenin. All these inhibitory mechanisms were suggested to suppress viral infection [71].

In a recent study by Dai et al. (2019), apigenin exhibited potent antiviral activity against *EV*-*A71* genotype C4 strain in vitro and in vivo. It was able to reduce the cytopathic effect to 50% at the concentration of 24.74 µM in 293S cells. Moreover, apigenin significantly reduced viral RNA and protein synthesis in vitro. When evaluated for its potential to protect newborn BALB/c mice from intracranial lethal challenge with *EV*-*A71*, apigenin was found to protect mice at the dose of 50 mg/Kg with 88.89% survival rate. Significant improvement in clinical scores and body mass were also observed in the treatment group as compared to the control group [72].

### 3.2. Baicalin

Baicalin is a flavone reported to inhibit the BrCr-Tr strain of *EV*-*A71* in post-infection assays with an IC_50_ of 4.96 μg/mL. Baicalin was shown to inhibit 75% viral infection at 8 h post-infection. Inhibition by baicalin at early stages of infection was due to a decrease in mRNA and protein levels of 3D polymerase. Moreover, it was also demonstrated that baicalin reduced the expressions of FasL and caspase-3, hence inhibiting *EV*-*A71* induced apoptosis in Rhabdomyosarcoma cells (RD). Additionally, it was identified to inhibit proinflammatory cytokines by the reduction in NF-κB p65 expression [73].

### 3.3. Chrysin and Its Derivative

Chrysin is a flavone widely found in leaves, fruits, and vegetables. Chrysin and its ester (diisopropyl chrysin-7-yl phosphate) were evaluated against the clinical isolate *EV*-*A71* Shzh-98 and were found to inhibit the viral replication process by blocking the activity of the 3C protein. Chrysin ester was found to be the more potent inhibitor of 3C protein than the parent chrysin. The activity was predicted in silico by simulation and was confirmed by in vitro protease inhibition assays. These flavonoids were able to decrease the production of the viral capsids, infectious virions and RNA of *EV*-*A71* [74].

### 3.4. Fisetin

Fisetin, a flavonol, was reported to possess antiviral activity against the clinical isolate *EV*-*A71* CMUH01 with an IC_50_ of 84.5 μM. Fisetin was able to inhibit the enzymatic activity of recombinant 3C protease of the virus in a cell-based assay. To further confirm its activity in vitro, antiviral assays were performed in RD cells. Fisetin was found to block the cleavage activity of 3C and thereby inhibited the viral replication [75].

### 3.5. Formononetin

The isoflavone formononetin was tested against several isolates of *EV*-*A71* including BrCr, H, JS-52 and Shzh 98. Inflammatory pathways such as ERK, p38, JNK, MAPK are usually upregulated in *EV*-*A71* infections and help in viral reproduction. Formononetin was shown to act on the MAPK pathway and reduced the activation of the downstream regulator of infection-induced inflammation mediators, prostaglandin E2 (PEG2) and cyclooxygenase 2 (COX2). This, in turn, reduced the viral infection. Moreover, it also suppressed the signaling cascade of the ERK, p38 and JNK pathways to bring down the infection [76].

In another study, formononetin was found to inhibit *EV*-*A71* strains G082, SH12-036 and SH12-276. Formononetin was able to inhibit attachment and entry of *EV*-*A71* in Vero cells. To further evaluate the exact target, the virus was passaged with increasing concentrations of formononetin and sequenced after the 10th passage. A common mutation causing K58T in VP4 was observed and this mutation was able to confer viral resistance towards formononetin. This resistance was further confirmed by the absence of K58T mutation in the wild type virus. The mutant virus was evaluated for attachment in Vero cells and the K58T mutation was found to inhibit viral attachment. It was hypothesized that formononetin could possibly cause an overall conformational change in the viral capsid [77].

Formononetin was also evaluated for its antiviral potential against *EV*-*A71* subgenotype C4 strain in 239S cells. It exhibited antiviral activity with an IC_50_ of 12.5 µM and significantly reduced viral replication and protein synthesis. It also protected newborn mice with a survival rate of 75% and significantly improved clinical score and animal body weight at a dose of 10 mg/Kg [72].

### 3.6. Hydroxyflavone and Its Derivatives

The hydroxyflavones are synthetic molecules, the core structure of which is also a backbone of flavonols. Wang et al. (2014) predicted that 7-hydroxyflavone and its phosphate ester diisopropyl-flavon7-yl phosphate were able to inhibit the activity of 3C protease of *EV*-*A71* in silico. They further evaluated in vitro potential of these flavonoids as antivirals against Shzh-98 isolate of *EV*-*A71*. It was found that 7-hydroxyflavone and diisopropyl-flavon7-yl phosphate both inhibited virus-induced cytopathic effect in RD cells with an IC_50_ of 23.45 and 13.63 μM, respectively. In addition, both flavonoids significantly reduced viral protein synthesis [78].

### 3.7. Kaempferol

The flavonol kaempferol was reported to inhibit the *EV*-*A71* clinical isolate Chuh-050530-5. Kaempferol treated cells were observed to reduce virus yield by 6 log units 24 hpi. Kaempferol was able to exert its action through induction of trans-factors such as the far upstream element-binding proteins (FUBP) and hnRPs. These trans-factors were identified to bind the highly conserved 5‘ UTR region of the virus genome and attenuated the viral infection. Moreover, it also disrupted the activity of IRES by an overall change in the composition of the trans-factors. This resulted in reduced viral infection by inhibition of translation and replication [79].

Kaempferol was found to inhibit *EV*-*A71* subgenotype C4 strain with an IC_50_ of 52.75 μM. It significantly reduced RNA copy number and protein synthesis 16 hpi. Newborn BALB/c mice were also protected from lethal challenge with the virus with 50 mg/Kg of kaempferol. The clinical score and body weight were found to be similar to that of the solvent treated group (negative control) [72].

### 3.8. Luteolin and Its Derivatives

Luteolin is one of the examples of flavonoids that acted at an early step of the *EV*-*A71* (subgenotype C4b) replication cycle by post-attachment inhibition of the virus. The IC_50_ was found to be 10 μM [55]. Moreover, when luteolin was tested against the prototype BrCr strain and the Fuyang0805 isolate of *EV*-*A71*, it was able to prevent virus-induced ROS generation, cellular apoptosis and inflammatory cytokine production such as IL-6 and CCL5 [71]. In another independent study, Dai et al. (2019) showed that luteolin exhibited similar inhibition patterns with an IC_50_ of 13.5 μM. It was identified to reduce viral replication and protein synthesis. Moreover, in the murine model, it showed 91.67% protection of newborn mice at a dose of 10 mg/Kg [72].

Eupafolin, a methoxy derivative of luteolin, was also found to inhibit *EV*-*A71* with an IC_50_ of 1.39 µM in an independent study. It was shown to inhibit IL-6 and chemokine RANTES which were upregulated during the in vitro viral infection. This inhibition resulted in the down-regulation of ERK, activated protein-1 (AP-1) and STAT3 mediated inflammatory signaling pathways [57].

### 3.9. Penduletin

Penduletin is a methylated flavonol abundantly found in the leaves of *Laggera pterodonta*. The compound was tested for its antiviral activity against *EV*-*A71* strain GZ-08-02. The flavonoid was a potent inhibitor of *EV*-*A71* with an IC_50_ of 0.17 and 0.20 µM in Vero and RD cells, respectively. Mechanistic studies revealed that penduletin specifically inhibited the production of infectious RNA viral progeny. Moreover, it was also able to reduce VP1 expression in post-infection assays, suggesting its ability to halt protein synthesis during viral replication process [80].

Penduletin also exhibited potent in vitro and in vivo antiviral activity against *EV*-*A71* subgenotype C4 strain. The IC_50_ was found to be 0.63 µM in 293 S cells and 66.67% newborn BALB/c mice survived the lethal challenge with *EV*-*A71* when mice were treated with 5 mg/Kg dose of penduletin [72].

### 3.10. Peracetate Pulicarine

Peracetate pulicarine is one of the less-studied flavonoids against viruses. It is a methoxyphenyl acetate, a derivative of a flavonoid which was found to possess anti-*EV*-*A71* activity in a recent screening of a flavonoid library. The antiviral activity of pulicarine was associated with inhibition of the 3D polymerase expression in *EV*-*A71* replication process [81].

### 3.11. Prunin

Prunin is a flavanone glycoside recently found to be potent antiviral against enteroviruses A and B including clinical isolates and strains of H, B5 and C4 genotypes of *EV*-*A71*. Prunin was able to suppress the activity of IRES and disrupted protein and RNA synthesis with an IC_50_ of 115.3 nM. It was further evaluated in the murine model using newborn BALB/C mice. Prunin was able to protect from the lethal challenge of *EV*-*A71* (strain 41) with a 100% survival rate at the doses of 3 mg/Kg and 10 mg/Kg per mice. Apart from suppression of IRES activity in vitro, prunin successfully protected mice from the infiltration of immune cells and inflammation of tissues. Moreover, prunin was also able to reduce viral antigen in hindlimb muscles of mice [68].

### 3.12. Quercetin and Its Derivatives

Quercetin has been found to possess broad-spectrum antiviral activity by several mechanisms. When tested against *EV*-*A71*, quercetin was able to target the post-attachment stage of *EV*-*A71* infection in vitro by disrupting viral RNA [55]. In another study, quercetin was predicted to interact with catalytic residues of the 3C protease and could inhibit the activity of viral the 3C protease in silico. To further confirm the findings, quercetin was evaluated for its antiviral activity using the SK-EV006/Malaysia/97 isolate of *EV*-*A71*. The IC_50_ of 8.8 μM was observed in Vero cells and 12.1 μM in RD cells. Quercetin was also investigated for its anti-apoptotic potential against virus-induced cell death. Quercetin was able to prevent the viral spread induced by apoptosis, thus making it a dual action antiviral agent [82].

Rutin is another glycoside flavonol derivative of quercetin. It was identified as antiviral against *EV*-*A71* using the recombinant 3C protease screening assay. To confirm its activity in vitro, rutin was tested against *EV*-*A71* strain CMUH01 (B5). Rutin was able to inhibit the replication stage of infection with an IC_50_ of 110 μM [75]. In another study, rutin was found to reduce the infectivity of the C4 subgenotype *EV*-*A71* with an IC_50_ of 200 μM. The anticipated mechanism was recognized as the suppression of the MEK1-ERK signaling pathway [83]. Direct inhibition of the 3C protease and interference with inflammatory pathways were assumed to contribute to the reduction of virus titres.

Quercetagetin, a synthetic derivative of quercetin, was identified to inhibit the RNA replication machinery and viral translation. The flavonoid was found to reduce the activity of the 3D polymerase in vitro [81]. Similarly, chrysosplenetin, a methylated derivative of quercetagetin, was identified to potently inhibit *EV*-*A71* by reducing the production of viral RNA and proteins [80].

Quercetin and its methoxy derivative isorhamnetin and chrysosplenetin were evaluated for their in vitro and in vivo potential as antivirals against *EV*-*A71* (subgenotype C4). Quercetin and chrysosplenetin displayed potent antiviral activities in vitro with an IC_50_ of 1.2 µM and 0.68 µM, respectively while isorhamnetin showed inhibition with an IC_50_ of 60.7 µM. All three flavonoids reduced RNA and protein synthesis in vitro. In the same study, these flavonoids were evaluated to identify their protective effect in mice from lethal challenge of *EV*-*A71*. Isorhamnetin was found to confer the best protection at the dose of 10 mg/Kg with 100% survival while quercetin could only protect 50% of the animals using the same dose. Despite being the most potent antiviral amongst the three flavonoids when tested in vitro, chrysosplenetin was the least effective in vivo. Only 30% of the animals survived after treatment with 5 mg/Kg of chrysosplenetin. All three flavonoids were able to improve the clinical score and bodyweight of the treated animals [72]. It should be taken into consideration that in this study, the route of virus inoculation was intracranial while flavonoids were administered by intraperitoneal (IP) route. This difference of injection routes could result in varying efficacy of flavonoids to cross the blood-brain barrier and potentially protect mice from virus. In vivo antiviral activity of different flavonoids against *EV*-*A71* has been summarized in Table 3.

### 3.13. Flavonoids Isolated from Scutellaria Baicalensis Georgi

Flavone derivatives such as mosloflavones, norwogonin and oroxylin A are abundant in *Scutellaria baicalensis Georgi*. These flavones were isolated as pure compounds and were reported to inhibit early stages of *EV*-*A71* replication with an IC_50_ of 37.72, 31.83 and 14.19 µg/mL, respectively. All three flavonoids were able to limit replication process, leading to inhibition of viral protein (VP2) synthesis. However, oroxylin was found to possess superior activity as compared to mosloflavones and norwogonin. Furthermore, these flavonoids were evaluated for their potential to inhibit viral attachment and entry. None of these flavonoids were found to be involved in the inhibition of attachment or entry of the virus in Vero cells [84].

### 3.14. Flavonoids with Unknown Mechanisms of Action against EV-A71

There are more examples of flavonoids that were reported to inhibit *EV*-*A71*. However, the mechanisms by which they modulated the infectivity of *EV*-*A71* are still unknown. Examples include nobiletin, morin hydrate, myricetin, taxifolin, diosmetin, dihydromyricetin [85], hesperidin [79], thio flavones [86] and galangin [55].

## 4. Structure–Activity Relationship among Potential Antiviral Flavonoids against *EV*-*A71*

**Figure 3:**
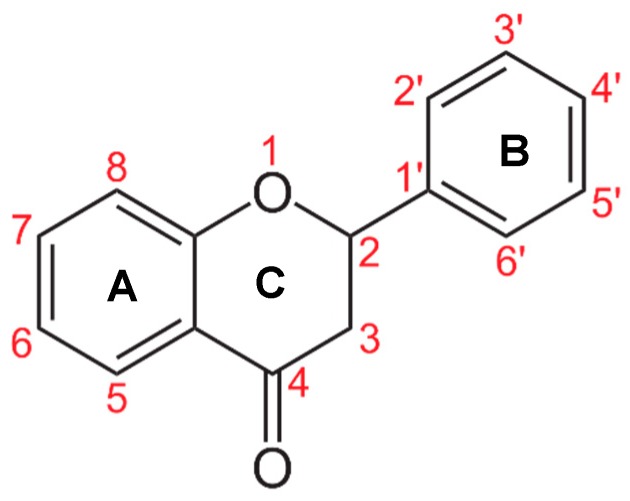
General chemical structure of flavone nucleus.

Flavonoid classes differ from each other by various substitutions particularly phenolic or hydroxyl (OH) groups on its flavan nucleus. Flavonoids that have successfully accounted for antiviral activity against *EV*-*A71* mostly belong to the flavonoid sub-classes flavonols and flavones. Flavones are dual fused aromatic rings (A and C) with an extension of a benzene ring (B) at position 2 of ring C (Figure 3) [87]. The skeleton “ring A fused with ring C” has previously reported to possess antiviral activity against the dengue virus NS2B-NS3 complex [88]. Moreover, hydroxylation on ring A, particularly on positions 5 and 7, was found to be favorable for antiviral activity against the influenza A virus [89]. Based on previous findings, it can be expected that the same structure could attribute towards antiviral activity against *EV*-*A71*.

It can be observed that the anti-*EV*-*A71* flavonoids with methylation have drastically improved the antiviral activity such as in the case of chrysosplenetin, eupafolin, penduletin and pruning (Table 4). Moreover, presence of methoxy substitution at the position 7 of ring A, along with hydroxyl or methoxy group at position 6 rendered higher activity with lowest IC_50_ values (chrysosplenetin, eupafolin, penduletin) amongst all evaluated flavonoids. Prunin, a glucoside of naringenin, having large glucose moiety at position 7 instead of methoxy substitution, interestingly showed potent antiviral activity as well. From these examples, it can be speculated that positions 6 and 7 of ring A require bulky group substitutions in order to possess potent activity against *EV*-*A71*. Possible interactions between different substitutions and *EV*-*A71* could be studied in silico to determine the nature of bonds involved in antiviral activity. It must be noted the antiviral activity of flavonoids was evaluated against specific *EV*-*A71* genotype/subgenotype. It is unknown if there is a broad spectrum of antiviral activities against other *EV*-*A71* genotypes/subgenotypes.

## 5. Limitations of Flavonoids as Antivirals against *EV*-*A71*

Flavonoids are abundant in fruits and vegetables and therefore are considered as safe and non-toxic for human consumption. However, the bioavailability of these flavonoids in their crude form is very poor. Use of isolated, purified or chemically synthesized small molecular weight flavonoids has increased prospects to reach the target. To further enhance the stability, solubility and improved systemic distribution, a nano-drug delivery system can be employed [90]. Despite possessing the potential to treat *EV*-*A71* in vitro and in mice, flavonoids have failed to reach clinical trials. Some of the possible reasons could include infancy of in vivo studies on flavonoids against *EV*-*A71* as there have been only two reports which were published in 2019, claiming absolute protection of mice by prunin and isorhamnetin [68,72]. Moreover, lack of long-term toxicity data on purified flavonoids contributes challenges to further progress these potential molecules to clinical trials. Another important factor could be the development of resistance by mutant viruses against these flavonoids. Like any other drug candidate, purified flavonoids require regulatory approval from the Food and Drug Administration (FDA) before it can be marketed as antivirals. Detailed pharmacokinetic, pharmacodynamics and long-term toxicology studies on these flavonoids are necessary before they can be offered as a therapeutic option.

## 6. Conclusions

Flavonoids such as penduletin, eupafolin, baicalin, luteolin, quercetin and chrysosplenetin have been shown to be antiviral agents against *EV*-*A71* based on their IC_50_ values (<10 µM). These flavonoids were also found to be safe and non-cytotoxic to human cells as indicated by CC_50_ values. Flavonoids such as prunin could confer 100% protection of neonatal mice while luteolin showed 91.67% protection against lethal challenges with *EV*-*A71*. However, flavonoids with higher IC_50_ values can also serve as a potential antiviral against *EV*-*A71*. For instance, kaempferol and isorhamnetin exhibited higher IC_50_ in vitro but both conferred significant protection of mice from viral challenge. The flavonoids that have not yet been tested in vivo could be further evaluated to identify their effectiveness.

Flavonoids, with their ability to target various stages of viral infection, are becoming a more focused topic to explore their potential as antivirals in the current era. Apart from their classical antioxidant properties, some flavonoids have been shown to inhibit viruses at the molecular level both in vitro and in vivo. In light of the current finding of the in vivo efficacy of some flavonoids against *EV*-*A71*, it can be concluded that flavonoids have great potential to be developed as therapeutic candidates against *EV*-*A71*. However, to make these flavonoids available as antivirals, a more in depth understanding of their pharmacological properties and clinical outcomes is warranted.

## Figures and Tables

**Table 1:** Examples of antiviral flavonoids against non-picornaviruses.

Flavonoid	Virus	Virus Family	Model	Stage of Virus Inhibition	Suggested Mechanism	Reference
**Apigenin**	*Hepatitis C virus (HCV)*	Flaviviridae	In vitro	Host factor modulation	Reduction in mature miRNA122	[12]
**Baicalin**	*Dengue virus*-*2 (DENV*-*2)*	Flaviviridae	In vitro	Attachment	Blockade of attachment of the virus to Vero cells	[14]
	*Human immunodeficiency virus*-*1 (HIV*-*1)*	Retroviridae	In vitro	Fusion	Inhibition of the fusion of virus envelope protein with T cells and monocytes expressing CD4/CXCR4 or CD4/CCR5	[13]
	*Influenza A virus*	Orthomyxoviridae	In vitro	Indirect: Immune-mediated infection control	Directly binds to NS1–p85β (RNA binding domain) to down-regulate IFN-ɣ and activates the JAK/STAT1 pathway that reduced the viral load	[15,16]
**Baicalein**	*Chikungunya virus (CHIKV)*	Togaviridae	In vitro	Prophylaxis	Inhibition of attachment by inhibiting extracellular particles such as nsP1, nsP3, and E2 proteins	[21]
	*DENV*-*2*	Flaviviridae	In silico	Replication	Binds to the NS3/NS2B and NS5 proteins	[18]
	*Japanese encephalitis virus (JEV)*	Flaviviridae	In vitro	Entry	Unknown Postulated to be an accumulation of the compound in cells to prevent entry or interaction with structural and/or non-structural protein(s)	[17]
**Epigallocatechin (ECG)**	*Influenza A and B viruses*	Orthomyxoviridae	In vitro	Replication	Acidification of the lysosomal and endosomal environment through clathrin-mediated endocytosis	[40]
**Epigallocatechin gallate (EGGC)**	*CHIKV*	Togaviridae	In vitro	Entry	Competitor for cellular co-receptors of target cells such as heparan sulfate or sialic acid	[43]
	*Epstein–Barr virus (EBV)*	Herpesviridae		Replication	Inhibition of *Zta*, *Rta* and *EA*-*D* genes by interrupting the MEK/ERK1/2 and PI3-K/Akt signaling pathway of the lytic cycle of a virus	[44]
	*Hepatitis B virus (HBV)*	Hepadnaviridae	In vitro	Replication	Impair the production of pre-core mRNA and replicative intermediates of DNA	[45]
					Acidification in lysosomes to make an unfavorable environment for virus replication	[47]
	*Herpes simplex virus (HSV)*	Herpesviridae	In vitro	Entry	Binds to glycoprotein B and D of virus	[42]
	*HIV*-*1*	Retroviridae	In vitro	Entry	Directly binds to CD4^+^ T-cells and blocks binding of envelope protein gp120 to cells	[41]
	*Zika virus*	Flaviviridae	In vitro	Entry	Interaction with the lipid envelope of virus	[46]
**Fisetin**	*CHIKV*	Togaviridae	In vitro	Replication	Inhibition of NS protein 1 and 3 and downregulation of E2 protein and its precursor pE2	[21]
	*DENV*-*2*	Flaviviridae	In vitro	Replication	Directly binds to the viral RNA to impede polymerases activity	[22]
**Genistein**	*HIV*	Retroviridae	In vitro	Assembly and release	Inhibition of Vpu protein involved in the formation of ion channels in infected cells	[19]
**Ginkgetin**	*Influenza A virus*	Orthomyxoviridae	In vitro	Assembly and release	Inhibition of sialidase	[20]
**Kaempferol**	*Coronavirus*	Coronaviridae	In vitro	Assembly and release	Inhibition of the release of progeny virus by blocking 3a channels of virus	[24]
	*Influenza A virus*	Orthomyxoviridae	In silico	Entry	Inhibition of neuraminidase enzyme	[23]
**Luteolin**	*Influenza A virus*	Orthomyxoviridae	In vitro	Entry	Interaction with hemagglutinins of virus	[25]
	*Severe acute respiratory syndrome coronavirus (SARS*-*CoV)*	Coronaviridae	In vitro	Entry	Binds to the S2 protein of virus	[25]
**Methoxyflavone, isoscutellarein, and 8-methoxy-isoscutellarein**	*Influenza A virus*	Orthomyxoviridae	In vitro	Early replication	Reduction in sialidase activity, lysosomal fusion and RNA polymerase activity	[27,48]
**Naringenin**	*CHIKV*	Togaviridae	In vitro	Replication	Reduction in RNA and proteins	[49]
	*DENV*-*2 and 4*	Flaviviridae	In vitro	Replication	Reduction in RNA levels	[29,30]
	*HCV*	Flaviviridae	In vitro	Replication	Inhibition of RNA and core protein	[28]
**Quercetin**	*HCV*	Flaviviridae	In vitro	Transcription	Inactivation of the NS3 helicase and NS5 protease	[8,9]
	*HSV*-*1, HSV*-*2, drug*-*resistant HSV*-*1*	Herpesviridae	In vitro	Binding and entry	N/R	[6]
	*Influenza A virus*	Orthomyxoviridae	In vitro	Binding and entry	Inhibition of neuraminidase activity by interaction with the viral subunit 2 of the hemagglutinin	[6]
	*Rabies virus*	Rhabdoviridae	In vivo	Cell protection	N/R	[3]
	*Vesicular stomatitis virus (VSV)*	Rhabdoviridae	In vivo	Indirect: Immune-mediated infection control	Activation of macrophages	[4]
**Quercetin 3-β-*O*-d-glucoside**	*Ebola virus*	Filoviridae	In vivo	Prophylaxis	Unknown	[10]
**Quercitrin**	*Influenza A virus*	Orthomyxoviridae	In vitro	Early replication	Reduction in mRNA synthesis	[7]
	*Rabies virus*	Rhabdoviridae	In vivo	Prophylaxis	Unknown	[3]
**Rutin (sulfated)**	*HIV*-*1*	Retroviridae	In vitro	Fusion	Inhibition of glycoprotein-mediated cell-cell fusion	[11]
	*HSV*	Herpesviridae	In vitro	Adsorption	Unknown	[11]
**Silibinin**	*HCV*	Flaviviridae	Phase II Clinical trial	Replication or immune-mediated infection control	Unknown. Postulated to be IFN-JAK/STAT independent immune-mediated antiviral mechanisms such as Regulation by interferon regulatory factor 3, Toll-like receptor 7 and p38 protein kinase pathways	[31]
**Silymarin**	*CHIKV*	Togaviridae	In vitro	Replication	Inhibition of viral proteins	[35]
	*DENV*-*2*	Flaviviridae	In silico	Replication	Inhibition of NS4B protein	[36]
	*EBV*	Herpesviridae	In vitro	Early antigen inactivation	N/R	[32]
	*HCV*	Flaviviridae	In vitro	Entry and fusion	Inhibition of viral pseudoparticles (pp) fusion with liposomes	[34]
	*Influenza A virus*	Orthomyxoviridae	In vitro	Late replication	Inhibition of viral mRNA synthesis	[37]
	*Mayaro virus*	Togaviridae	In vitro	Replication	Inhibition of reactive oxygen species (ROS) and reduction in levels of malondialdehyde (MDA)	[33]
**Tea catechins**	*Influenza A virus*	Orthomyxoviridae	In vitro	Adsorption and entry	Interaction with hemagglutinins of virus	[38]
**Tetra-*O*-methyl quercetin**	*Influenza A virus*	Orthomyxoviridae	In vitro	Entry	Interaction with mannose-rich hemagglutinin domains of virus	[5]

N/R = not reported, NS = nonstructural. Structures of flavonoids are provided in Supplementary Materials Table S1.

**Table 2:** Examples of antiviral flavonoids against picornaviruses.

Flavonoid	Picornavirus	Model	Stage of Virus Inhibition	Suggested Mechanism	Reference
**3-Methylquercetin**	*Poliovirus*	In vitro	Late replication	Blocked of genomic RNA synthesis	[51]
				Reduction in viral protein and RNA synthesis	[52]
**3-Methylkaempferol**	*Poliovirus*-*1*	In vitro	Replication	Inhibition of positive-strand of viral RNA	[58]
**5,3′-Dihydroxy-3,6,7,8,4′-pentamethoxyflavone and 5-hydroxy-3,6,7,3′,4′-pentamethoxyflavone**	*Poliovirus*-*1*	In vitro	Replication	Postulated to be inhibition of cellular processes (apoptosis and downstream signaling pathways)	[67]
**5,7,4′-Trihydroxy-3′-methoxyflavone**	*Rhinovirus* *(HRV)*	In silico	Entry	Inhibition by binding to human rhinovirus protein grid	[61]
**6-Chloro-4′-oxazolinylflavanone**	*Poliovirus*-*2*	In vitro	Replication	N/R	[63]
	*HRV*-*1B*	In vitro	Replication	N/R	[63]
**7-*O*-galloyltricetifavan and 7,4′-di-Ogalloyltricetifavan**	*Coxsackievirus B3* *(CV*-*B3)*	In vitro	N/R	N/R	[64]
**Chrysosplenol C**	*Poliovirus*	In vitro	Replication	N/R	[65]
**Desmanthin-1**	*Coxsackieviruses A16* *(CV*-*A16)*	In vitro	Replication	N/R	[66]
**Dihydroquercetin**	*Coxsackievirus B4* *(CV*-*B4)*	In vivo	Indirect: Immune-mediated infection control	Reduction in viral immune mediators (ROS-mediated signaling and oxidative stress	[54]
**Epigallocatechin-3-Gallate**	*Poliovirus*-*1*	In vitro	Virucidal effect (irreversible)	N/R	[60]
**Eupafolin**	*CV*-*A16*	In vitro	Attachment	Reduction in IL-6 and RANTES and inactivation of downstream signaling pathways (ERK1/2, c-Jun, and STAT3)	[57]
**Kaempferol-3-*O*-[2″,6″-di-*O*-Z-p-coumaroyl]-β-d-glucopyranoside and derivatives**	*CV*-*B3*	In vitro	Replication	N/R	[59]
	*HRV*-*1B*	In vitro	Replication	N/R	[59]
**Luteolin**	*CV*-*A16*	In vitro	Replication	Inhibition of viral RNA synthesis	[55]
	*Poliovirus*	In vitro	Replication	N/R	[56]
**Myricitrin**	*CV*-*A16*	In vitro	Replication	N/R	[66]
**Pachypodol (RO 09-0179)**	*CV*	In vitro	Early replication	Interference with viral replications between the uncoating and RNA synthesis stage	[53]
	*Poliovirus*	In vitro	Late replication	Blocked the synthesis of positive-strand RNA	[51]
	*HRV*	In vitro	Early replication	Interference with viral replications between the uncoating and RNA synthesis stage	[53]
**Prunin**	*Enteroviruses A and B*	In vitro and in vivo	Translation and replication	Inhibition of IRES activity and protein synthesis	[68]
**Quercetin**	*Encephalomyocarditis virus (EMCV)*	In vivo	Indirect: Immune-mediated infection control	Activation of macrophages	[4]
	*Mengo virus*	In vivo	Indirect: Immune-mediated infection control	Activation of macrophages	[4]
	*HRV*	In vitro	Transcription and translation	Reduction in endocytosis of virus and phosphorylation of Akt (effector of phosphoinositol 3-kinase). Repression of interferon and interleukin-8 response resulted in lower viral RNA and capsid protein production.	[50]
	*HRV*	In vivo	Indirect: Immune mediated infection control	Suppression of viral immune mediators	[50]
**RO 09-0298**	*CV*-*B1*	In vivo	N/R	N/R	[53]
**Sakuranetin**	*HRV*-*3*	In vitro	Replication	Antioxidant activity through inhibition of viral adsorption	[62]

Structures of flavonoids are provided in Supplementary Materials Table S1.

**Table 3:** Antiviral activity of flavonoids against *Enterovirus A71* in newborn mice.

Flavonoid	In Vitro EC_50_ (µM)	Lethal Dose of Challenge Virus	In Vivo Dose of Flavonoid	Survival Rate	Duration of Treatment	Reference
**Apigenin**	24.74	600,000 TCID_50_	50 mg/Kg	88.89%	Once a day for 7 days, starting from 2 h post-infection	[72]
**Chrysosplenetin**	0.68	600,000 TCID_50_	5 and 1 mg/Kg	30%	Once a day for 7 days, starting from 2 h post-infection	[72]
**Formononetin**	12.5	600,000 TCID_50_	10 mg/Kg	75%	Once a day for 7 days, starting from 2 h post-infection	[72]
**Isorhamnetin**	60.7	600,000 TCID_50_	10 mg/Kg	100%	Once a day for 7 days, starting from 2 h post-infection	[72]
**Kaempferol**	52.75	600,000 TCID_50_	50 mg/Kg	88.89%	Once a day for 7 days, starting from 2 h post-infection	[72]
**Luteolin**	13.5	600,000 TCID_50_	10 mg/Kg	91.67%	Once a day for 7 days, starting from 2 h post-infection	[72]
**Penduletin**	0.63	600,000 TCID_50_	5 mg/Kg	66.67%	Once a day for 7 days, starting from 2 h post-infection	[72]
**Prunin**	0.115	2 × 10^7^ PFU	3 and 10 mg/Kg	100%	Once a day for 7 days, starting from 1 or 6 h post infection	[68]
**Quercetin**	1.2	600,000 TCID_50_	10 mg/Kg	50%	Once a day for 7 days, starting from 2 h post infection	[72]

Note: Flavonoids were administered into the newborn mice by the intraperitoneal route.

**Table 4:** In vitro antiviral activities of flavonoids against Enterovirus A71.

Flavonoid	Structure	*EV*-*A71* Strain * (Genotype/Subgenotype)	Antiviral Activity/IC_50_	Cytoxicity/CC_50_	Reference
**Apigenin**	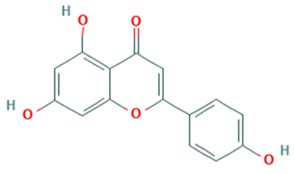	Fuyang 0805 (C4a)	10.3 μM	79.0 μM (RD cells)	[70]
		Fuyang0805 (C4a) BrCr (A)	Not reported	>200 μM (RD and Vero cells)	[71]
**Baicalin**	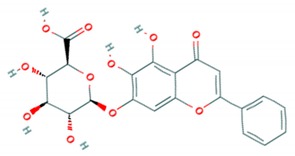	BrCr (A)	4.96 μg/mL	823.53 µg/mL (RD cells)	[73]
**Chrysin** **Ester of chrysin (CR)**	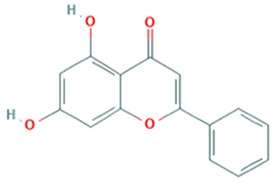	SHZH-98 (C4)	C = 13.86 μM	>200 μM (RD cells)	[74]
			CR = 24.12 μM	>200 μM (RD cells)	
**Chrysosplenetin**	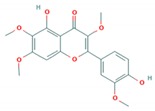	GZ-08-02 (Accession # FJ360545)	0.17 μM (Vero cells)	18.27 μM (Vero cells)	[80]
			0.20 μM (RD cells)	13.90 μM (RD cells)	
**Eupafolin**	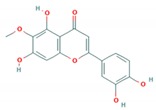	Not reported	0.44 µg/mL (RD cells)	355.87 µg/mL (RD cells)	[57]
**Fisetin**	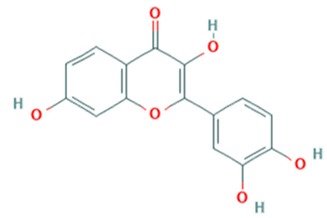	CMUH01 (B5)	85 μM	>1000 μM (RD cells)	[75]
**Formononetin**	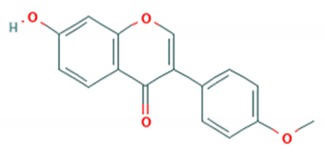	SHZH-98 (C4) JS-52 (C4) H BrCr (A)	3.45–3.95 μM 17.87 ± 8.51 μM 11.11 ± 9.23 μM 6.47 ± 4.40 μM	149.38 μM (Vero cells) 198.80 μM (SK-N-SH cells)	[76]
**Galangin**	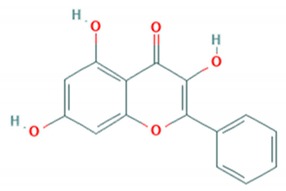	C4b	Not reported	Not reported	[55]
**Hesperetin**	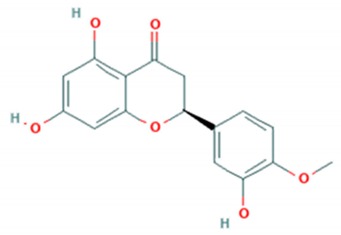	Cmuh-050530-5 (Accession # HM807310)	Not reported	>50 μM (RD cells)	[79]
**Hesperidin**	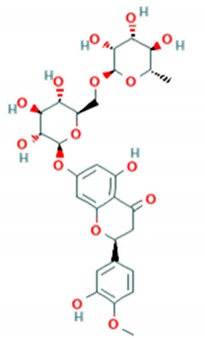	Cmuh-050530-5 (Accession # HM807310)	Not reported	>50 μM (RD cells)	[79]
**Hydroxyflavone (HF) and its phosphate ester**	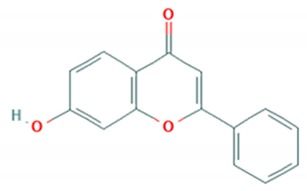	SHZH-98 (C4)	23.45 μM	>200 μM (RD cells)	[78]
	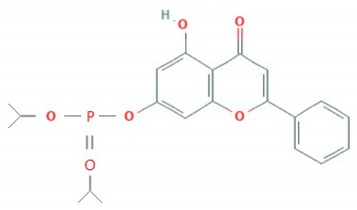	SHZH-98 (C4)	13.63 μM	>200 μM (RD cells)	[78]
**Kaempferol**	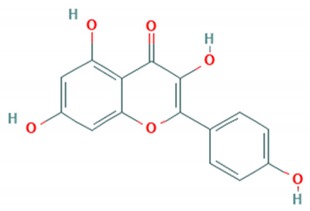	Cmuh-050530-5 (Accession # HM807310)	Not reported 6 log reduction at 24 hrs	>50 μM (RD cells)	[79]
**Luteolin**	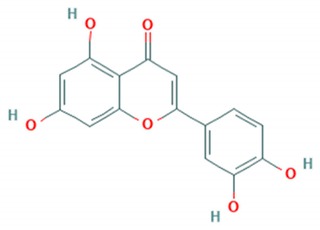	C4b	10 μM	148.02 μM (RDS cells)	[55]
				292.00 μM (RD cells)	
		Fuyang0805 (C4a) BrCr (A)	Not reported	178.65 μM (Vero cells)	[71]
				157 μM (Vero cells)	
				200 μM (RD cells)	
**Penduletin**	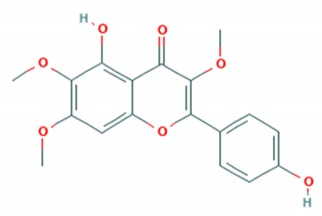	GZ-08-02 (Accession # FJ360545)	0.17 µM (Vero cells)	111.46 µM (Vero cells)	[80]
			0.37 µM (RD cells)	74.18 µM (RD cells)	
**Peracetate pulicarine**	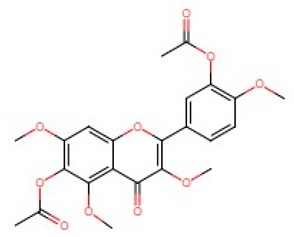	5865/sin/000009 (B4)	Not reported.	>20 µg/mL (RD cells)	[81]
		5511-SIN-00 (B5)	2.5 Log reduction		
**Prunin**	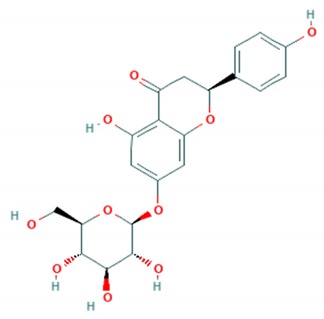	*EV*-*A71* clinical isolates, *EV*-*A71* strains H, B5 and C4 genotypes	115.3 nM	2715 nM (RD cells)	[68]
**Quercetagetin**	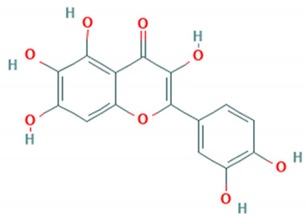	5865/sin/000009 (B4)	Not reported.	>20 µg/mL (RD cells)	[81]
		5511-SIN-00 (B5)	3.5 Log reduction		
**Quercetin**	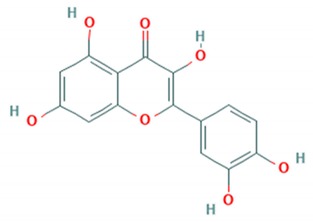	SK-EV006/Malaysia/97 (Accession # AB469182)	12.1 μM (RD cells)	>200 μM (RD and Vero cells)	[82]
			8.8 μM (Vero cells)		
**Rutin**	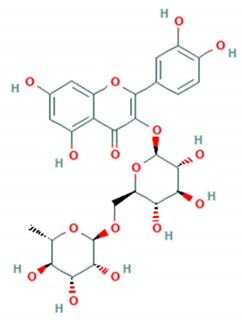	CMUH01 (B5)	110 μM	>1000 μM (RD cells)	[75]
**Thio flavones** **(Multiple)** **4b** **7d** **7i** **8b** **9b**	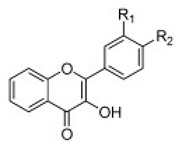 4b = R1 = OCH_3_, R2 = H 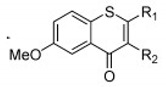 7d = R1 = 4-methoxyphenyl, R2 = H 7i = R1 = CH_3_, R2 = Cl 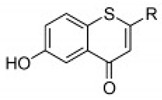 8b = R = n-propyl 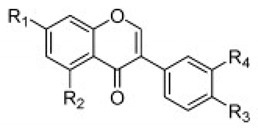 9b = R1 = OH, R2 = H, R3 = OH, R4 = OH	SHZH-98 (C4)	4b = 16.9 μM	4d = 29.23 μM	[86]
			7d = 8.27 μM	7d = 107.34 μM	
			7i = 39.63 μM	7i = 133.15 μM	
			8b = 100.86 μM	8b = 174.41 μM	
			9b = 5.48 μM	9b = 23.75 μM (Vero cells)	

* Accession number is mentioned where genotype/subgenotype is not reported; IC_50_ is the inhibitory concentration of flavonoid required to cause 50% inhibition of virus; CC_50_ is the cytotoxic concentration of flavonoid required to cause 50% cell death.

## Supplementary Materials

The following are available online at https://www.mdpi.com/1999-4915/12/2/184/s1, Table S1: Molecular structures of antiviral flavonoids. Structures of flavonoids discussed in Tables 1 and 2 are provided in supplementary Table S1.
