# Correction: Lalani, S. and Poh, C.L. Flavonoids as Antiviral Agents for *Enterovirus A71* (*EV-A71*). *Viruses* 2020, *12*, 184

**DOI:** 10.3390/v12070712

**Published:** 2020-06-30

**Authors:** Salima Lalani, Chit Laa Poh

**Affiliations:** Centre for Virus and Vaccine Research, Sunway University, Bandar Sunway, Subang Jaya, Selangor 47500, Malaysia; salimalalani@msn.com

## 1. Change in Table/Figure

We have recently been made aware by Mr. Saravanan (National University of Singapore) that the structure of prunin flavonoid used in their study was different to the one that we reported. Therefore, the authors wish to make the following corrections to this paper [1]:

Replace the structure of prunin reported in Table 4 on page 25 and the Supplementary Table.


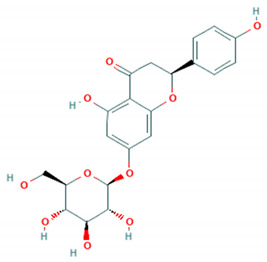


with this structure, provided by the inventor:


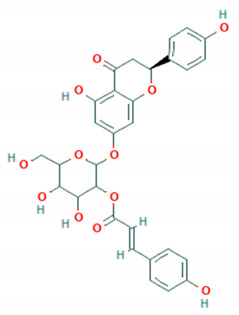


## 2. Change in Main Text of the Body

There is a typographical error in the name of prunin (please see page 18, Section 4, paragraph 2) [1]. The current paragraph reads as

It can be observed that the anti-EV-A71 flavonoids with methylation have drastically improved the antiviral activity such as in the case of chrysosplenetin, eupafolin, penduletin and pruning (Table 4).

We would like to correct the spelling of prunin as below.

It can be observed that the anti-EV-A71 flavonoids with methylation have drastically improved the antiviral activity, such as in the case of chrysosplenetin, eupafolin, penduletin and prunin (Table 4).

The authors would like to apologize for any inconvenience caused to readers by these changes.
