# Supplementary material for: Flavonoids as Antiviral Agents for Enterovirus A71 (EV-A71)
Source: Viruses. 2020 Feb 6;12(2):184. doi: 10.3390/v12020184 (PMC7077323; doi:10.3390/v12020184)
Supplement: Supplementary file 1 [file viruses-12-00184-s001.pdf]

Supplementary Table S1: Molecular structures of antiviral flavonoids

**Flavonoid**	Structure
**Apigenin**	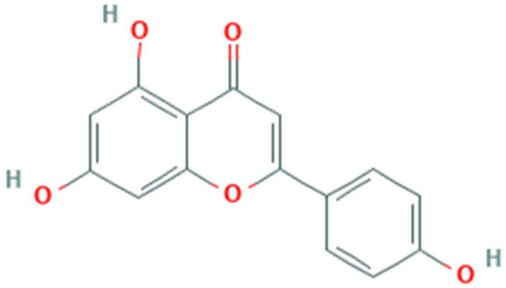
**Baicalin**	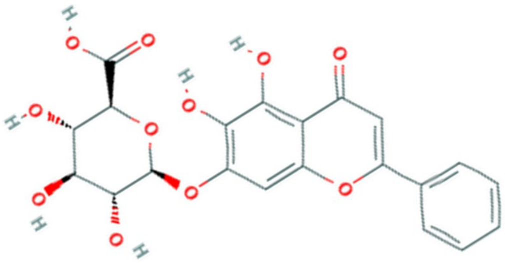
**Baicalein**	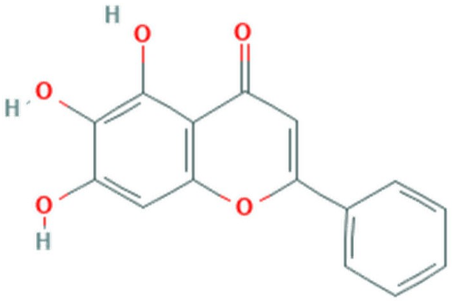
**6-Chloro-4′-oxazolinylflavanone**	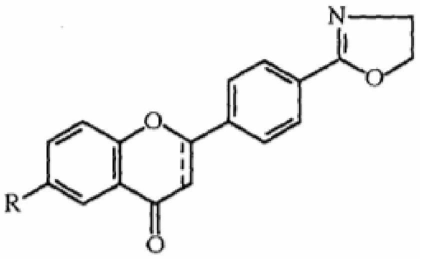 R=Cl

**Chrysosplenol C**	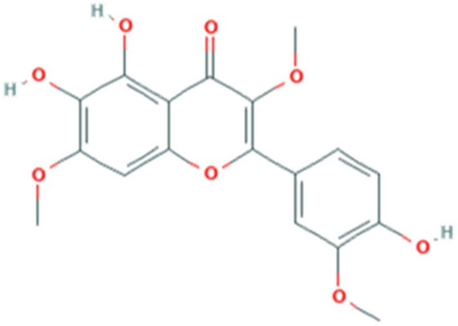
**Desmanthin-1**	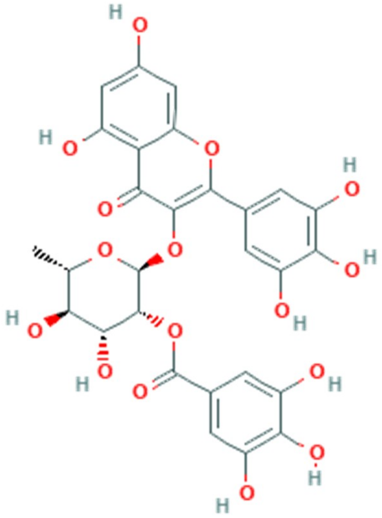
**Dihydroquercetin**	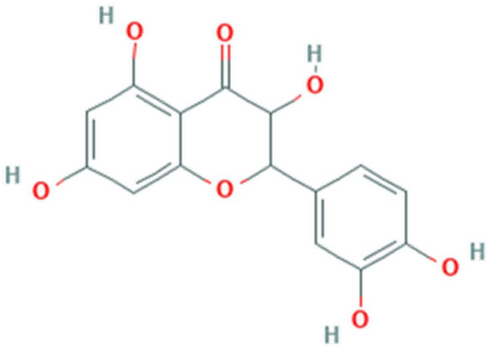
**5,3'-Dihydroxy-3,6,7,8,4'-pentamethoxyflavone**	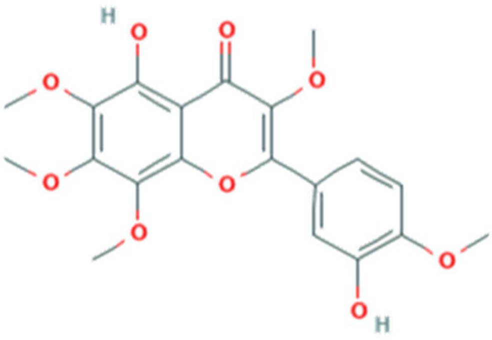

**7,4′-Di-O-galloyltricetifavan**	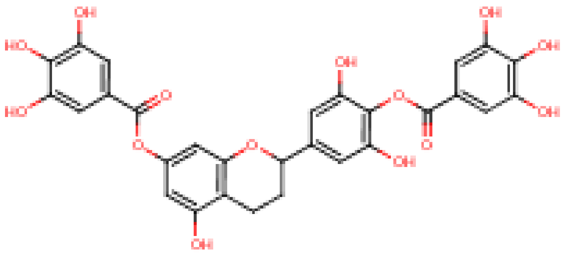
**Epigallocatechin (ECG)**	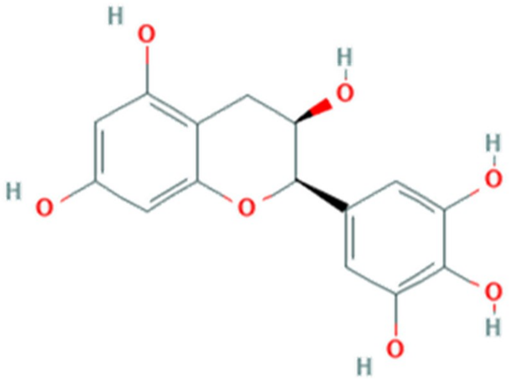
**Epigallocatechin gallate (EGGC)**	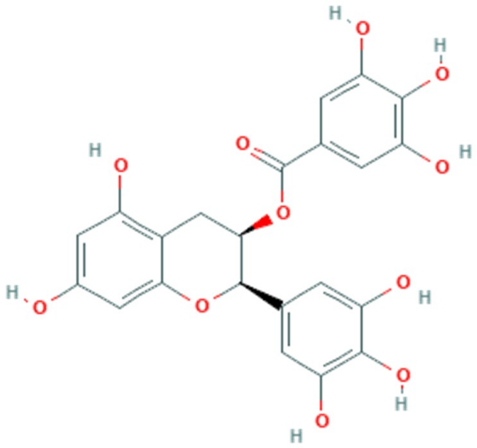
**Eupafolin**	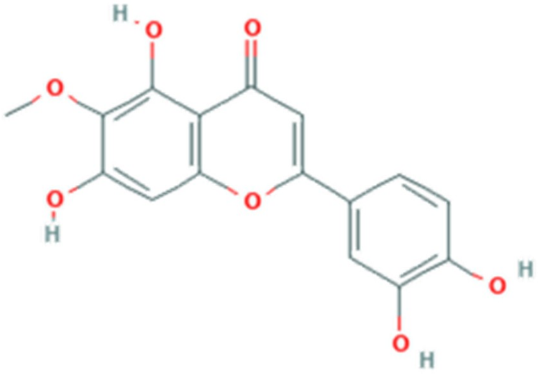

**Fisetin**	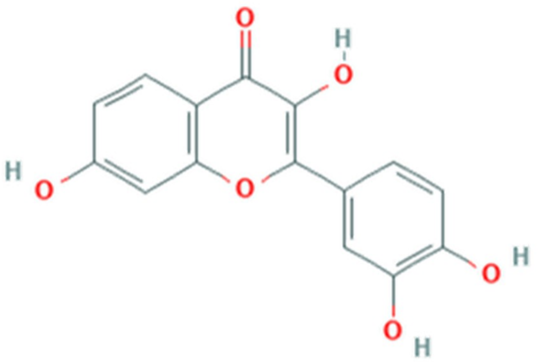
**Genistein**	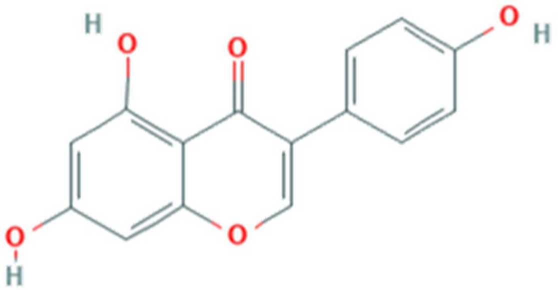
**Ginkgetin**	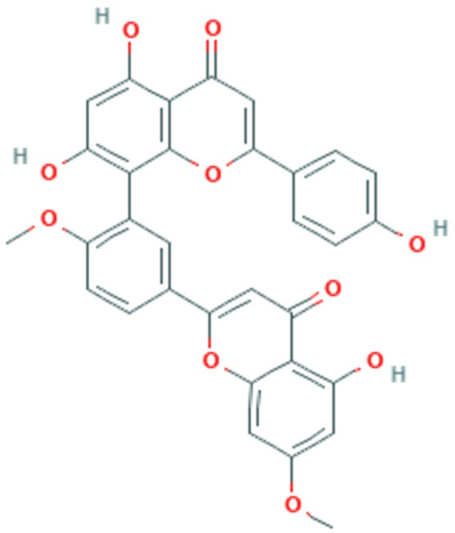
**5-hydroxy-3,6,7,3',4'-pentamethoxyflavone**	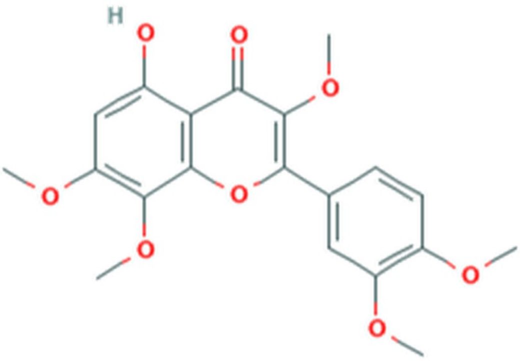

**Isoscutellarein**	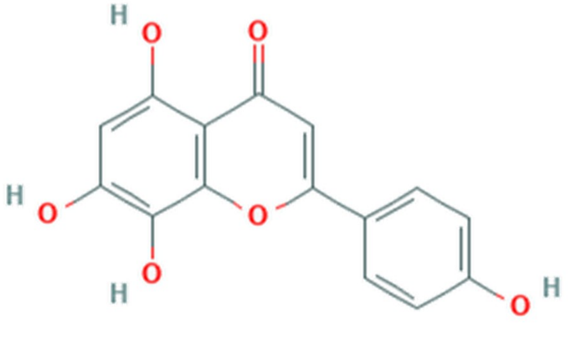
**Kaempferol**	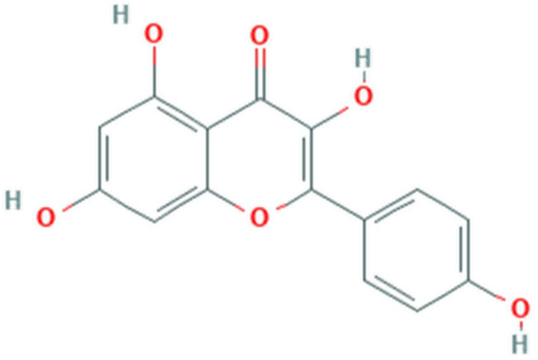
**Kaempferol-3-O-[2″,6″-di-O-Z-p-coumaroyl]-β-D-glucopyranoside**	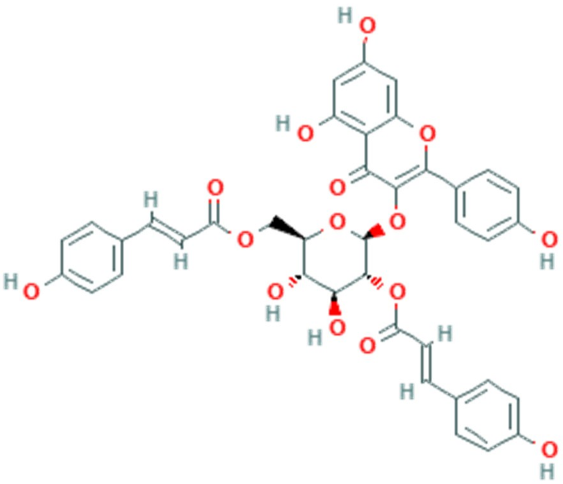
**Luteolin**	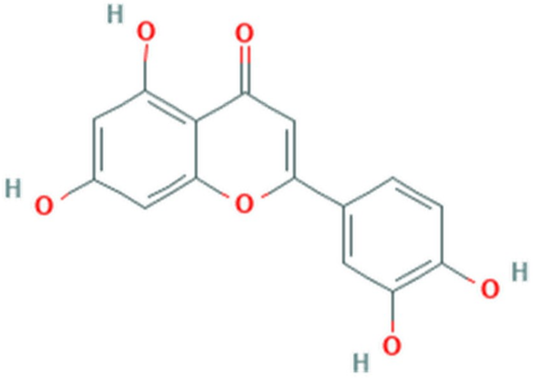

**Methoxyflavone**	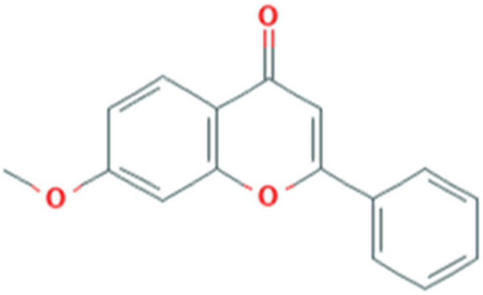
**8-Methoxy-isoscutellarein**	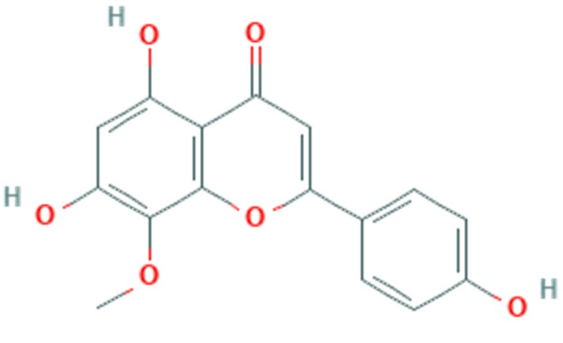
**3-Methylkaempferol**	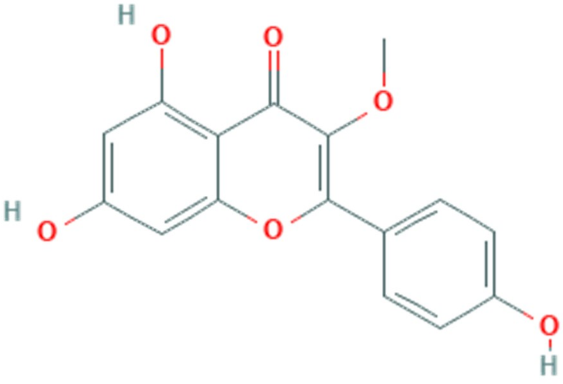
**3-Methylquercetin**	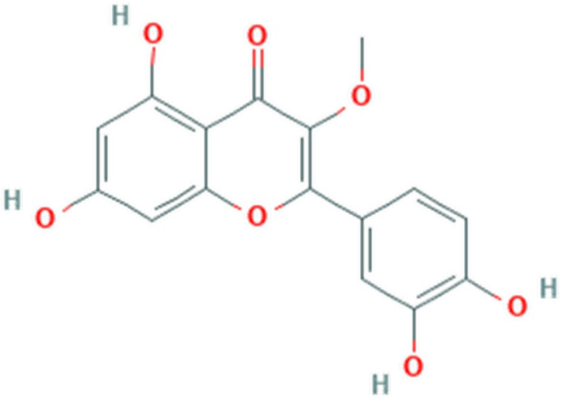

**Myricitrin**	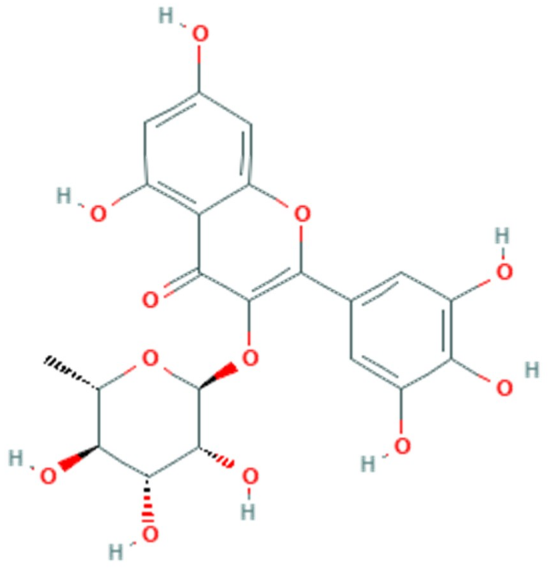
**Naringenin**	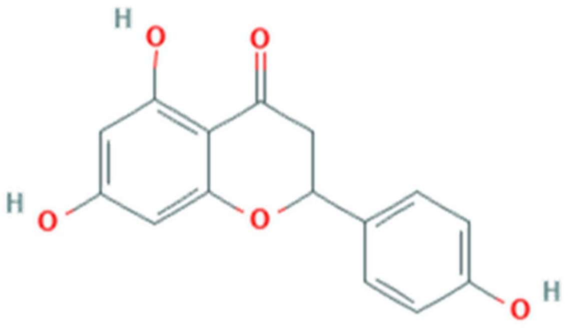
**7-O-galloyltricetifavan**	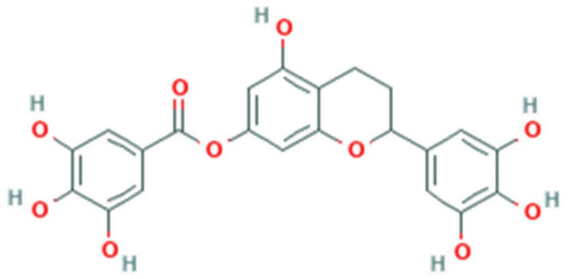
**Pachypodol (RO 09-0179)**	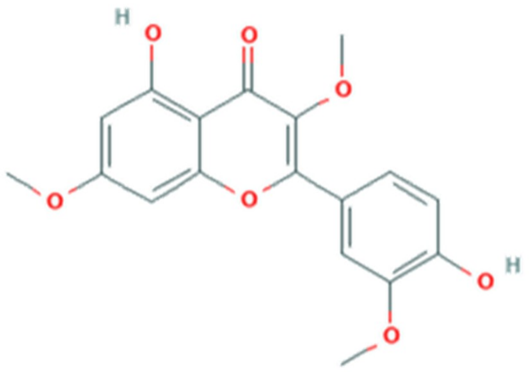

**Prunin**	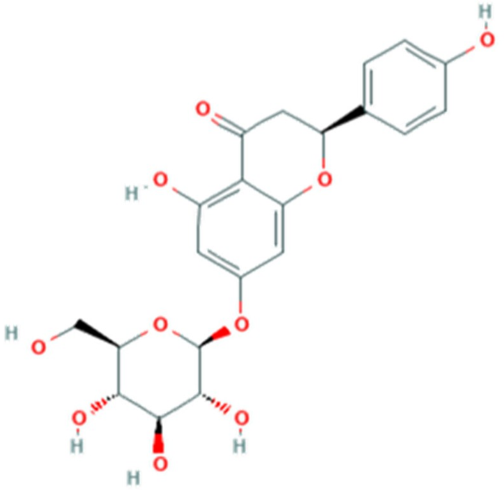
**Quercetin**	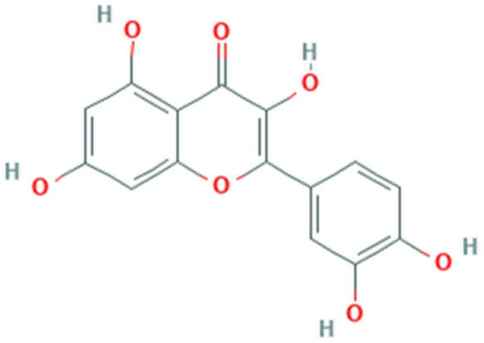
**Quercetin 3-β-O-D-glucoside**	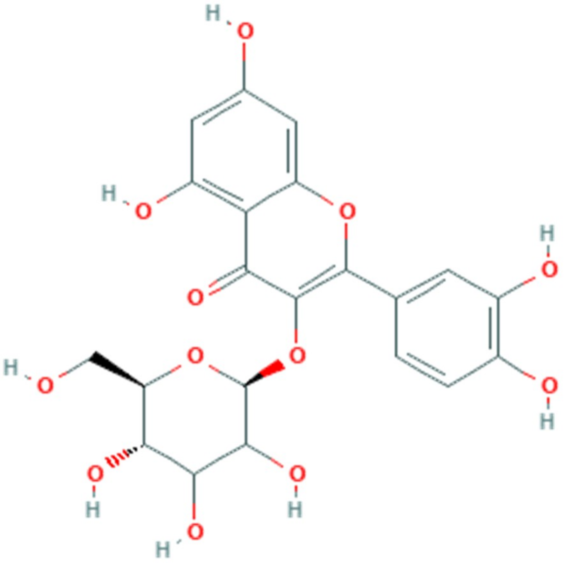

**Quercitrin**	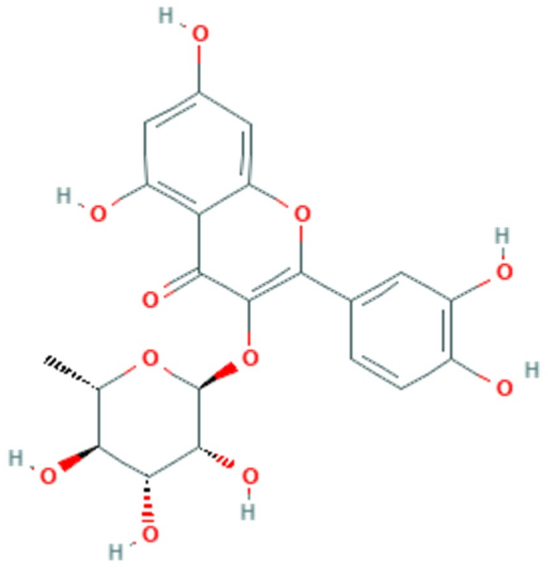
**RO 09-0298**	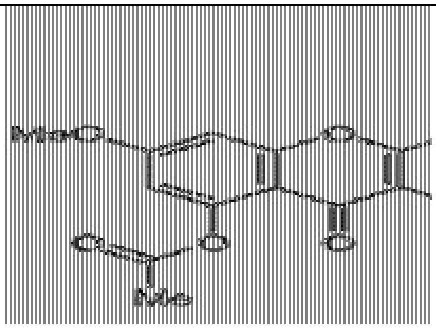
**Rutin**	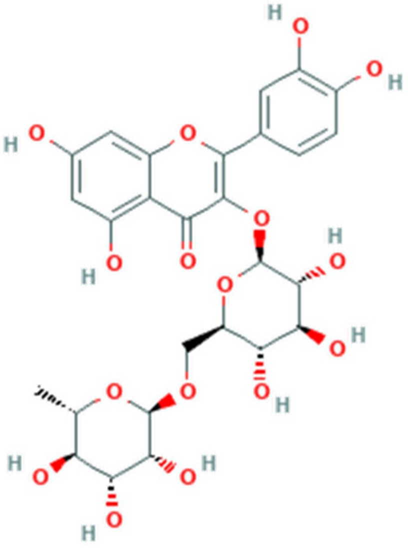

**Sakuranetin**	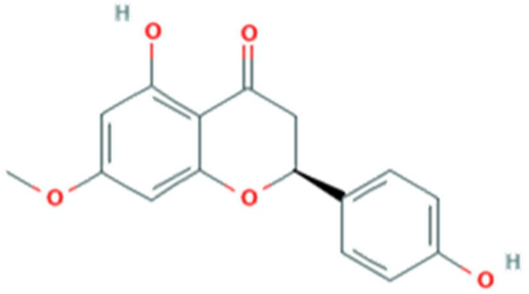
**Silibinin**	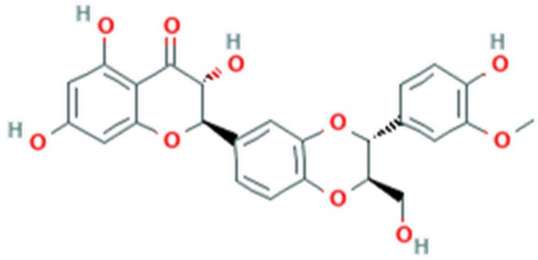
**Silymarin**	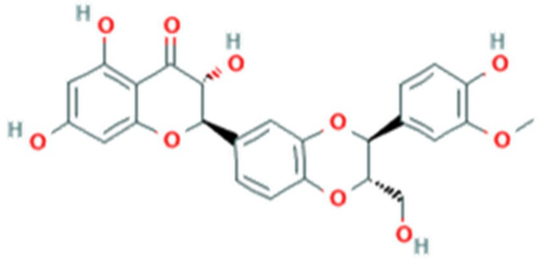
**5,7,4′-Trihydroxy-3′-methoxyflavone**	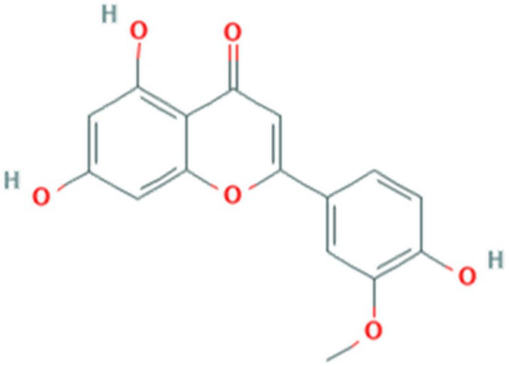
**Tetra-O-methyl quercetin**	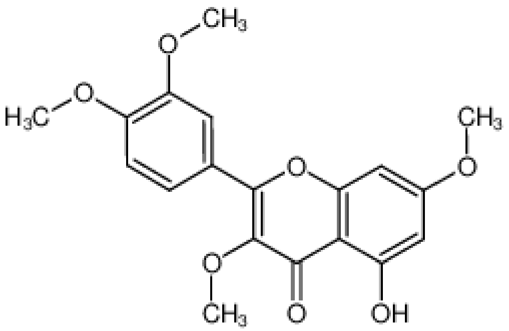
